# Effects of water and nitrogen coupling on the photosynthetic characteristics, yield, and quality of *Isatis indigotica*

**DOI:** 10.1038/s41598-021-96747-0

**Published:** 2021-08-30

**Authors:** Yucai Wang, Xiucheng He, Fuqiang Li, Haoliang Deng, Zeyi Wang, Caixia Huang, Yi Han, Yuchun Ba, Lian Lei, Changlong Zhang

**Affiliations:** 1 College of Water Conservancy and Hydropower Engineering, Gansu Agricultural University, 1 Yingmen Village, Lanzhou, 730000 Gansu Province China; 2 College of Civil Engineering, Hexi University, Zhangye, 734000 China; 3 Yimin Irrigation Experimental Station, Minle County, Zhangye, 734500 China

**Keywords:** Ecology, Plant sciences

## Abstract

*Isatis indigotica* is a commercial medicinal crop that is widely cultivated with high water and nutrient application, in the arid areas of northwest China. Rational irrigation and nitrogen application are key factors for successful crop management. The objective of this study was to determine the effect of water and nitrogen coupling on the photosynthetic characteristics, yield, and quality of *Isatis indigotica* produced in northwestern China. Field trials were conducted for 2 consecutive years at an irrigation test station. Data on photosynthetic parameters, yield, and quality were collected from individual *Isatis indigotica* for each treatment during 2018–2019. The application of nitrogen significantly increased photosynthetic rates and yield under the same irrigation conditions. However, the yields were reduced in the excess water treatments (W3N1 and W3N2) and in the excess nitrogen treatments (W1N3, W2N3, and W3N3) in contrast to the optimum W2N2 treatment. Moreover, the quality indicators of the W2N2 treatment decreased compared with CK, which was due to water stress and more photoassimilates being available to the roots, but the effective quality index value could be effectively improved by greatly increasing the yield.

## Introduction

In the oasis region of Hexi, China, agricultural development is limited by water shortages and the excessive application of nitrogen fertilizer. The major focus of research is determining ways to improve water use efficiency and to reduce nitrogen application. Two essential factors for crop growth are water and fertilizer application. Rational irrigation and fertilization can effectively improve crop yield. The main problem facing modern agriculture is how to promote fertilizer with water and transfer water with fertilizer^1^. Reasonable water–nitrogen coupling optimization models have received increasing attention from researchers. Water is the medium through which soil nutrients are effectively absorbed by plants, improving the efficiency of nitrogen utilization. However, excessive water application will lead to leaching and the loss of nitrogen, while excessive nitrogen application will lead to non-point source pollution^2^.

At present, an increasing number of studies have focused on the effects of coupled water and nitrogen application on crops. For example, Liu et al.^3^, Li et al.^4^, Sui et al.^5^, Fiasconaro et al.^6^, and Gholamhoseini et al.^7^ studied the effects of the coupling of water and nitrogen on the yield and quality of rice, tomato, cotton, alfalfa, and corn, respectively. The synergistic effect of water and nitrogen can save water, increase yield, and improve crop quality effectively. The yield and water use efficiency of *Isatis indigotica* were not high due to flood irrigation. Moreover, as growers seek to increase yields, the phenomenon of excessive nitrogen application is very common^8^.

Scholars have studied the effect of water on the yield and quality of *Isatis indigotica* from the aspects of water-saving irrigation systems^9^ and water stress^10^. However, no research is currently available on the influence of water and nitrogen fertilizer on photosynthesis, yield, and quality. Local farmers apply fertilizers and irrigate relying only on empirical observations, resulting in low-efficiency water and fertilizer utilization. Therefore, the aim of this research was to study the effect of water and nitrogen coupling on the photosynthesis, yield, and quality of *Isatis indigotica*.

## Materials and methods

### Location

The experiments were carried out at the Yimin Irrigation Pilot Station (Gansu, China; 100° 43′ E, 38° 39′ N) in the middle reaches of the Flood River Irrigation District, Minle County, Gansu Province from May to October in 2018 and 2019. The Yimin Irrigation Experimental Station and Flood River Administration Office, Minle County, China provided permission to collect *Isatis indigotica*. The experimental zone has a continental desert steppe climate, with a dry climate, abundant heat, abundant light, and little rain; the altitude is about 1970 m. According to the yearly precipitation data, the average annual precipitation in this area is 215 mm with little precipitation and large variation. The contradiction between supply and demand is prominent, and drought is frequent. The soil is light loam with a pH value of 7.22, the field water-holding capacity of tillage layer soil is 24%, the soil bulk density is 1.4 g cm^−3^, the groundwater level is low, and the area does not exhibit salinization or alkalization.

### Test materials and cultivation methods

*Isatis indigotica* seeds were cultivated and provided in the Department of Chinese herbal medicine, Gansu Agricultural University. The seed purity was 96%, the weight per 1000 seeds was 9.873 g, the germination rate was 87.6%, and the germination potential was 46.4%. The seeds were sown on May 3 and harvested on October 13. The seeds were sown at 30.0 kg hm^−2^ and the planting density was 800,000 plants hm^−2^. The plant spacing of *Isatis indigotica* was about 20 cm, and the row spacing was about 30 cm. Before sowing, the experimental zone was ploughed for 30 cm to remove weeds manually. Moreover, 350 kg hm^−2^ calcium superphosphate (12% P_2_O_5_, 10% S, and 16% Ca) and 200 kg hm^−2^ source potassium (25% K_2_O) were applied. Each experimental plot was separated by a film with a width of 60 cm to prevent underground water seepage.

### Experimental design

The growth of *Isatis indigotica* is divided into four stages based on the growth characteristics: the seedling stage, vegetative stage, fleshy root growth stage, and fleshy root maturity. Three irrigation treatments were set in the field experiment. Treatments W1, W2, and W3 were 60–70% of field water capacity, 70–80% of field water capacity, and 80–90% of field water capacity, respectively. There were three nitrogen treatments: N1: 150 kg hm^−2^, N2: 200 kg hm^−2^, and N3: 250 kg hm^−2^. There were 10 water control treatments, of which CK was the control treatment. Each treatment was repeated three times, with a total of 30 plots. The area of each plot was 36 m^2^ (9 m × 4 m). The method of irrigation was drip irrigation under mulch. The specific experimental design is presented in Table 1. All the experiments on plants were carried out in accordance with guidelines of Gansu Agricultural University.

**Table 1:** Experimental treatment of water–nitrogen coupling in *Isatis indigotica*.

Treatment	Serial number	N application/(kg hm^−2^)	Field water capacity (%)
Low water, low nitrogen	W1N1	150	60–70
Low water, medium nitrogen	W1N2	200	60–70
Low water, high nitrogen	W1N3	250	60–70
Medium water, low nitrogen	W2N1	150	70–80
Medium water, medium nitrogen	W2N2	200	70–80
Medium water, high nitrogen	W2N3	250	70–80
High water, low nitrogen	W3N1	150	80–90
High water, medium nitrogen	W3N2	200	80–90
High water, high nitrogen	W3N3	250	80–90
Control treatment	W0N0	0	0

### Photosynthetic characteristics

Photosynthesis was measured using a Li-6400 portable photosynthesis system (Zhangye, Gansu, China) during the period of 9:30–10:30 a.m. on May 15, June 1, June 20, July 10, and July 25 in 2018 and 2019. The physiological parameters of net photosynthetic rate (Pn), stomatal conductance (Gs), transpiration rate (Tr), and intercellular CO_2_ concentration (Ci) were measured in situ for the seventh or eighth leaf that was fully expanded^11^ (counted back from the apex of new shoots). In each treatment, for each sampled plant, 3–4 sunlit healthy leaves were randomly selected from different parts of the plant and labeled; each leaf was measured once, in triplicate for each treatment. The average value of each treatment was calculated.

### Yield and water use efficiency

At harvest time, the yield of each plot was weighed and counted separately, and the yield of each treatment was the average of three replicates. Water use efficiency (WUE) was calculated as follows:

$$WUE = Y/ET_{a} ,$$

where *WUE* is the water use efficiency (kg hm^−2^ mm), *Y* is the yield per unit area of *Isatis indigotica* (kg hm^−2^ mm), and *ET*_*a*_ is the water consumption (mm) during the whole growth period of *Isatis indigotica*.

### Quality

Determination of indigo, indirubin, and (R, S)-goitrin content was performed as follows: the Chinese Pharmacopoeia method^12^ was used to extract indigo, indirubin, and (R, S)-goitrin, and their contents were determined by high-performance liquid chromatography. The content of polysaccharide in *Isatis indigotica* root was determined by phenol–sulfuric acid colorimetry.

### Statistical analysis

**Figure 1:**
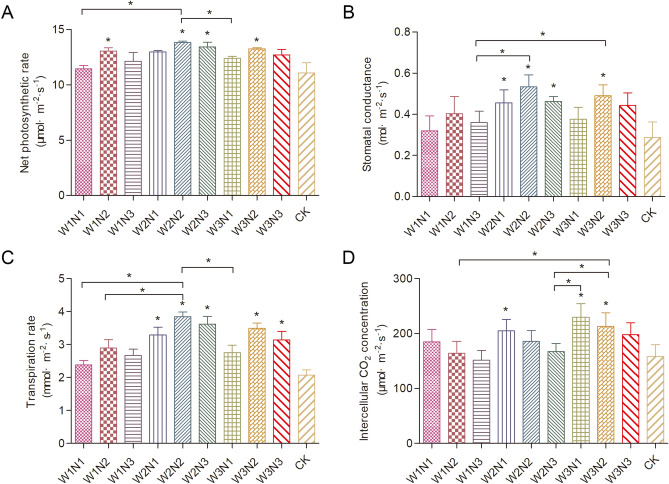
Photosynthetic characteristic values for *Isatis indigotica* in all treatments. The values shown are the mean ± SD, n = 3. Asterisks (*) indicate a significant difference at the P ≤ 0.05 level.

**Figure 2:**
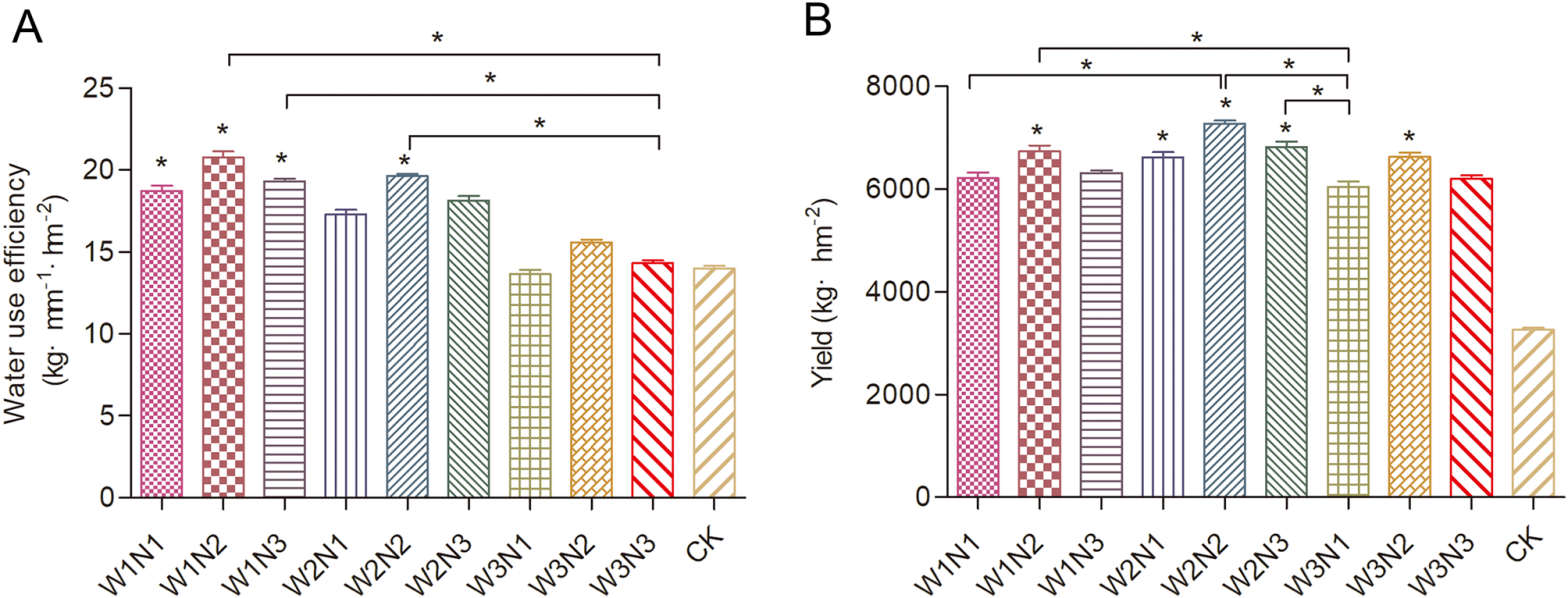
Yield and water use efficiency values for *Isatis indigotica* in all treatments. The values shown are the mean ± SD, n = 3. Asterisks (*) indicate a significant difference at the P ≤ 0.05 level.

**Figure 3:**
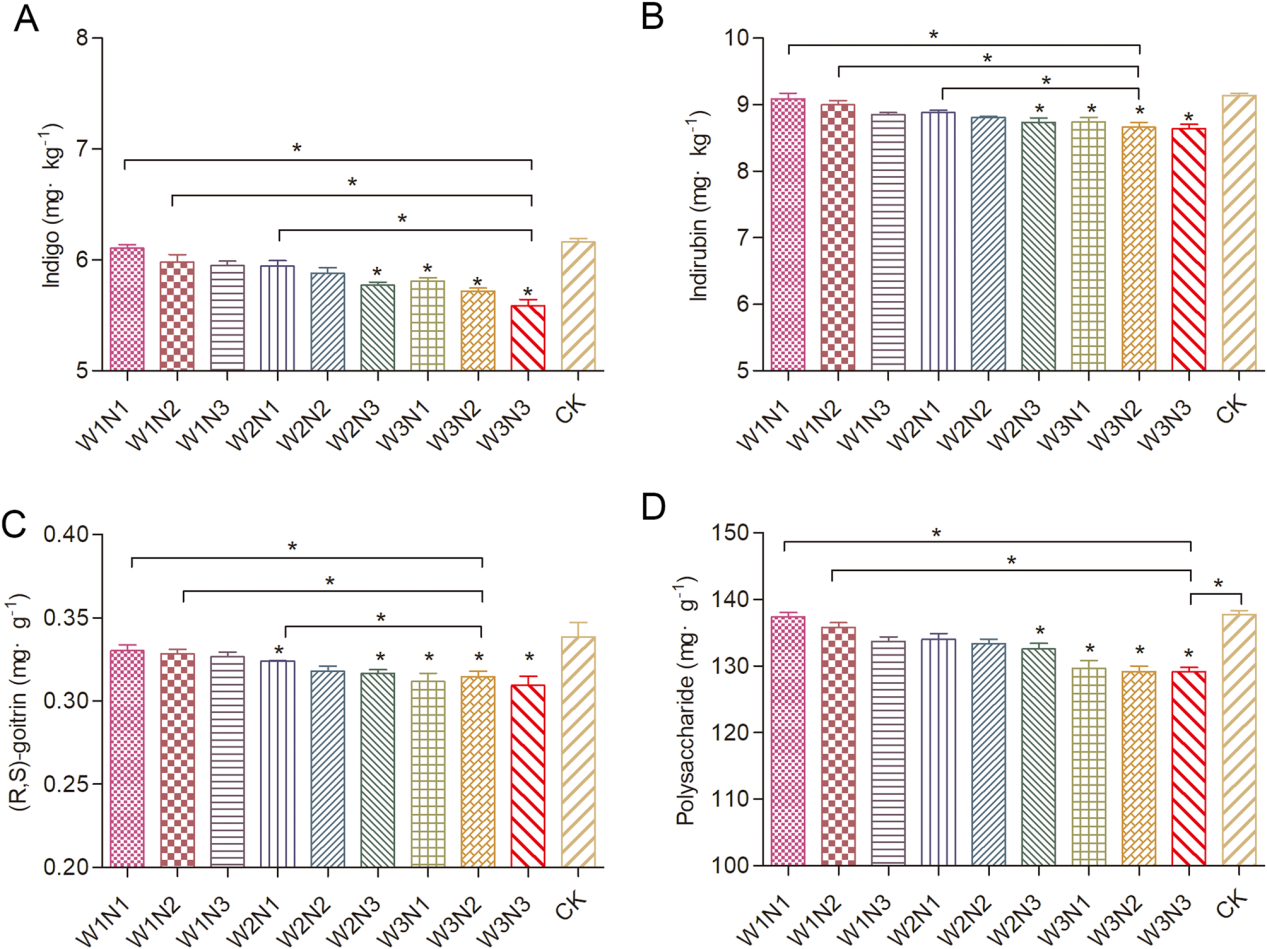
Quality values for *Isatis indigotica* in all treatments. The values shown are the mean ± SD, n = 3. Asterisks (*) indicate a significant difference at the P ≤ 0.05 level.

The data analyses were performed using SPSS software package (Version 21.0, IBM Corp., Armonk, NY). The significance of differences between treatments for the different measured parameters was evaluated using independent samples, followed by the Kruskal–Wallis test (P ≤ 0.05). Figures 1, 2 and 3 were all generated by GraphPad Prism software (5.0, GraphPad Software., San Diego, California). The data in each table were average values of three replicates.

## Results and discussion

### Photosynthetic characteristics

Water and nitrogen coupling treatment had a significant effect on the photosynthetic characteristics (Fig. 1). Generally, the net photosynthetic rates of the treatments were in the following order: CK, W1N1, W1N3, W3N1, W3N3, W2N1, W1N2, W3N2, W2N3, and W2N2. The treatments with low water and low nitrogen had significantly lower net photosynthetic rates than W2N2. The stomatal conductance and transpiration rate changed in similar patterns. The net photosynthetic rate showed a unimodal trend with the increase of nitrogen application at the same irrigation level. Under the same nitrogen application level, the net photosynthetic rate increased first and then decreased slowly with the increase of irrigation amount, with the highest photosynthetic rates in the order of W2 > W3 > W1. The net photosynthetic rate was the highest, with a mean value was 13.87 μmol m^−2^ s^−1^, in treatment W2N2. The results showed that severe water stress and excessive nitrogen were not conducive to the absorption and utilization of water and nutrients by crop roots, which led to the decrease of the photosynthetic rate. The effect of water and nitrogen treatment on the intercellular CO_2_ concentration was significant (Fig. 1). Under the condition of excessive water or nitrogen, the photosynthesis of *Isatis indigotica* decreased, and the intercellular CO_2_ concentration showed a trend opposite to that of the net photosynthetic rate.

Compared with N, P, and K deficiency treatments, water–N coupling could increase the Pn of crops, which was the same as that of other fruit trees and vegetables^13^. Accumulated photoassimilates in the third internode of the upper part of the main stems, as well as in the flag leaf sheath, are mobilized in a higher proportion and can contribute to grain filling in rice plants subjected to water stress in the tillering phase^14^. The Pn, Gs, and Tr of maize leaves at the seedling stage decreased significantly, while the Ci increased significantly when the nitrogen application rate was low^15^.

The experiments with *Isatis indigotica* demonstrate that the Pn, Gs, and Tr under the same irrigation level first increased and then decreased with the increase of the nitrogen application rate. The net photosynthetic rate, transpiration rate, and stomatal conductance of *Isatis indigotica* were improved by rational nitrogen application. Studies have reported similar findings in *Isatis indigotica*; *w*ith the decrease of N level, the net photosynthetic rate, transpiration rate, and stomatal conductance of leaves gradually decreased, while the intercellular CO_2_ concentration increased^16,17^. Under reasonable water and nitrogen coordination conditions, the synergistic effect of water and nitrogen increased, which effectively promoted the photosynthesis of *Isatis indigotica*. Under the condition of too much nitrogen or too little water, the antagonism of water and nitrogen was obvious, and the photosynthesis of *Isatis indigotica* was inhibited to a certain extent.

### Yield and water use efficiency

The *Isatis indigotica* yield values presented are the average of two consecutive years of water–nitrogen trials (Fig. 2). The *I. indigotica* yields differed significantly between the water–nitrogen treatments; the W2N2 and W2N3 treatments had the highest yields at 7277.5 and 6820.5 kg hm^−2^, respectively. The lowest yield of 3264.5 kg hm^−2^ was recorded in the control treatment. The yields of all treatments were significantly higher than that of the control treatment. The yields of the W2N2 and W2N3 treatments were significantly higher than those of the W1N1 and the W3N1 treatments. With the increase of the nitrogen application rate, the yield first increased and then decreased under the same irrigation conditions.

The water use efficiency values of *Isatis indigotica* presented are the average of 2 consecutive years of water–nitrogen trials (Fig. 2). The water use efficiency of *Isatis indigotica* differed significantly between the water–nitrogen treatments; the W1N2 and W2N2 treatments had the highest yields at 20.78 and 19.63 kg mm^−1^ hm^−2^, respectively. The lowest yield of 13.65 kg mm^−1^ hm^−2^ was recorded in the W3N1 treatment. The water use efficiency values of the W1N2 and W2N2 treatments were significantly higher than that of the W3N3 treatment, which was the treatment with excess water and nitrogen fertilizer. The water use efficiency decreased with the increase of irrigation under the same nitrogen application conditions. The water use efficiency first increased and then decreased with the increase in nitrogen application rate under the same irrigation conditions. The W2N2 treatment had the highest yield and water use efficiency. Therefore, the water–nitrogen coupling mode of medium water and medium nitrogen application achieved the highest yield and effectively saved water. This was mainly due to the moderate water and nitrogen to promote the photosynthesis of *Isatis indigotica* and lead to more dry matter accumulation, so as to increase the yield.

Generally, appropriate water deficits can improve crop yield and water use efficiency^18,19^, and rational fertilization can increase crop yield, such as in fruit trees and vegetables^20–22^. The yield increase in the current experiment was probably related to reasonable water stress and reasonable nitrogen application; the W2N2 treatment had the highest yield and water use efficiency. However, excessive water and nitrogen reduced the yield and water use efficiency of *Isatis indigotica*. This was consistent with recent research reports^23,24^. Compared with the local flooding irrigation and excessive nitrogen fertilizer mode, the W2N2 treatment with moderate water and nitrogen application not only obtained a high yield but also significantly improved the water use efficiency. This method could reduce the effect of excessive water and fertilizer application on soil productivity and would be a better water and nitrogen management model for local *Isatis indigotica* production.

### Quality

The *Isatis indigotica* quality values presented are the average of two consecutive years of water–nitrogen trials (Fig. 3). These quality indicators mainly include the following content indicators: indigo, indirubin, (R, S)-goitrin, and polysaccharides. The *Isatis indigotica* quality indicators differed significantly between the water–nitrogen treatments. The CK treatment had the highest values of all quality indicators. Each quality indicator decreased gradually with the increase of water content under the same nitrogen application conditions. Each quality indicator decreased gradually with the increase of nitrogen application under the same water conditions. The (R, S)-goitrin content of the W2N2 treatment decreased by 6.5% compared with CK and by 3.9% compared with the W1N1 treatment.

Water is the medium for improving crop quality. Generally, the crop quality was improved by a suitable water deficit^25–27^ and reasonable fertilization^28–30^. The quality of *Isatis indigotica* in the current experiment increased gradually with the decrease of water. The water deficit treatment increased the content of effective components and improved the quality of *Isatis indigotica*. The content of the effective components in all treatments reached the pharmacopoeia standard^12^. The quality indicator values of each treatment in the current experiment were significantly lower than those of the CK treatment, but there was little difference in the quality indicator values between each treatment. Moreover, the yield of the control treatment was much lower than that of other treatments. Therefore, the effective quality content of the control treatment was lower than other treatments. Excessive water and nitrogen inputs were not conducive to quality, which was not consistent with recent research reports^31^. Generally, the water-nitrogen coupling type of W_2_N_60_ was antagonism basing on the average yield of winter wheat in the 10 years^32^. Some scholars have studied the irrigation of jujube that WUE and ANUE of jujube cannot reach the maximum at the same time. Different ratio of water and nitrogen will produce coupling and antagonism^33^. The results showed that total N applications over 200 kg ha^−1^ did not increase yield or quality. Water deficit treatment could be increased the content of effective components and improve the quality of *Isatis indigotica*. Due to the high evaporation, moderate water stress and effective use of nitrogen fertilizer, the active components of *Isatis indigotica* were easier to accumulate in its roots. The synergistic effect of water and nitrogen will lead to the accumulation of active components in *Isatis indigotica.*

## Conclusions

The findings of this study indicate that water–nitrogen coupling has distinct benefits. The *Isatis indigotica* yields differed significantly between the water–nitrogen treatments; the W2N2 and W2N3 treatments had the highest yields at 7277.5 and 6820.5 kg hm^−2^, respectively. The W2N2 treatment of water and nitrogen at medium levels significantly promoted net photosynthetic rates and increased the yield and water use efficiency. However, the lack or excess of water and nitrogen would greatly reduce these benefits. Reasonable water stress had a significant positive impact on the quality of *Isatis indigotica* under the same nitrogen application conditions but no significant effect on the yield. The yield and quality of *Isatis indigotica* could be improved by reasonable water stress and moderate nitrogen application.
